# Compounds Identification in Semen Cuscutae by Ultra-High-Performance Liquid Chromatography (UPLCs) Coupled to Electrospray Ionization Mass Spectrometry

**DOI:** 10.3390/molecules23051199

**Published:** 2018-05-17

**Authors:** Ying Zhang, Hui Xiong, Xinfang Xu, Xue Xue, Mengnan Liu, Shuya Xu, Huan Liu, Yan Gao, Hui Zhang, Xiangri Li

**Affiliations:** School of Chinese Materia Medica, Beijing University of Chinese Medicine, Beijing 102488, China; zhang0312ying@163.com (Y.Z.); xionghui@bucm.edu.cn (H.X.); xuxinfang007@163.com (X.X.); sherry.xue@bucm.edu.cn (X.X.); 20160931810@bucm.edu.cn (M.L.); xushuya11@163.com (S.X.); 20150931937@bucm.edu.cn (H.L.); 20150931752@bucm.edu.cn (Y.G.); zh19930503@sina.com (H.Z.)

**Keywords:** Semen Cuscutae, ultra-high-performance liquid chromatography coupled to electrospray ionization mass spectrometry, chlorogenic acids, flavonoids

## Abstract

Semen Cuscutae is commonly used in traditional Chinese medicine and contains a series of compounds such as flavonoids, chlorogenic acids and lignans. In this study, we identified different kinds of compositions by ultra-high-performance liquid chromatography (UPLC) coupled to electrospray ionization mass spectrometry (MS). A total of 45 compounds were observed, including 20 chlorogenic acids, 23 flavonoids and 2 lignans. 23 of them are reported for the first time including 6-*O*-caffeoyl-*β*-glucose, 3-*O*-(4′-*O*-Caffeoylglucosyl) quinic acid, etc. Their structures were established by retention behavior, extensive analyses of their MS spectra and further determined by comparison of their MS data with those reported in the literature. As chlorogenic acids and flavonoids are phenolic compounds that are predominant in Semen Cuscutae, in conclusion, phenolic compounds are the major constituents of Semen Cuscutae.

## 1. Introduction

Semen Cuscutae is the dry mature seed of *Cuscuta australis* R.Br. or *Cuscuta chinensis* Lam., belonging to convolvulaceae family. It was first recorded in the “*Shen Nong’s Herbal*” as an upper grade drug about 2000 years ago. Semen Cuscutae has been widely prescribed by Chinese medicinal practitioners to nourish the liver and kidney, improve eyesight, treat the aching and weakness of the loins and knees, prevent abortion, and treat diarrhea due to hypofunction of the kidney and the spleen [1]. Previous phytochemical investigations on Semen Cuscutae have led to the isolation of a series of natural compounds, including flavonoids, lignans, polysaccharides, alkaloids and other chemicals [2,3,4].

Most studies of identification and quantification of flavonoids and polysaccharide in Semen Cuscutae have been performed by HPLC-UV [5,6,7], but few studies have been performed by ultra-high-performance liquid chromatography (UPLC) coupled with electrospray ionization tandem mass spectrometry. This method has the advantage that it is more sensitive and selective than a HPLC-UV, leading to a more exact identification of a higher number of compounds [8].

The purpose of this work was to identify different kinds of ingredients with significant biological functions in Semen Cuscutae for further phytochemical and pharmacological study.

## 2. Results and Discussion

### 2.1. Optimization of UPLC-MS Conditions

**Figure 1:**
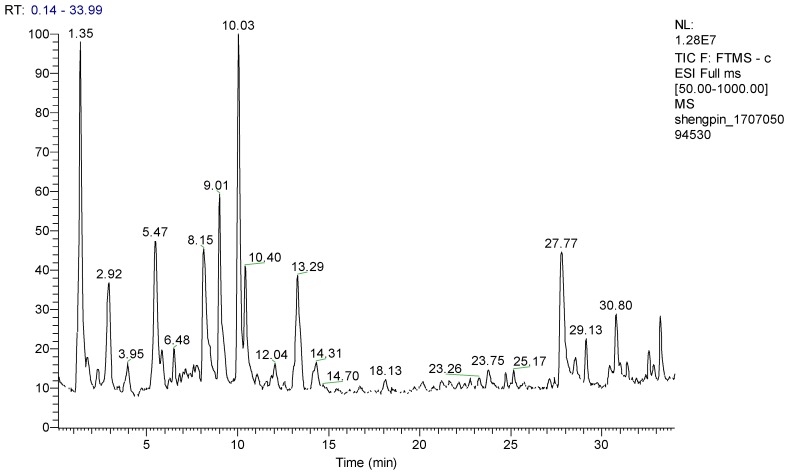
The total ion chromatographic (TIC) of Semen Cuscutae

In this study, an optimized chromatographic separation was achieved using acetonitrile-water containing 0.05% formic acid solvent system as the mobile phases. Waters ACQUITY UPLC BEH C_18_ (2.1 × 100 mm i.d., 1.7 μm) was selected for qualitative analysis due to better separation efficiency. A representative total ion chromatographic (TIC) were shown in Figure 1.

To obtain the satisfactory analytical method, chromatographic conditions, including mobile phase (methanol, acetonitrile and acetonitrile-water), flow rate (0.1, 0.2, and 0.3 mL·min^−1^), formic acid addition (0.05% and 0.1%), and column type (Waters ACQUITY BEH C_18_, 2.1 × 100 mm, 1.7 μm, and Agilent Eclipse Plus C_18_ column (2.1 × 100 mm i.d., 1.8 μm) were optimized after several trials. Meanwhile, in order to achieve massive fragment ions, all the factors related to MS performance, including ionization mode, sheath gas flow rate, aux gas flow rate, spray voltage of the ion source, and collision energy have been optimized.

### 2.2. Optimization for Sample Extraction

The extraction method had been established by our team [5]. The best extracted condition was established as follows: 1.0 g of sample was extracted by refluxing using 50 mL 80% methanol as solvent for 2 h. To obtain satisfactory extraction efficiency, the extraction method (refluxing and ultrasonication), extraction concentration (40%, 60% and 80%), and extraction time (0.5, 1 and 2 h) were optimized.

### 2.3. Structural Characterization by UPLC-MS

**Figure 2:**
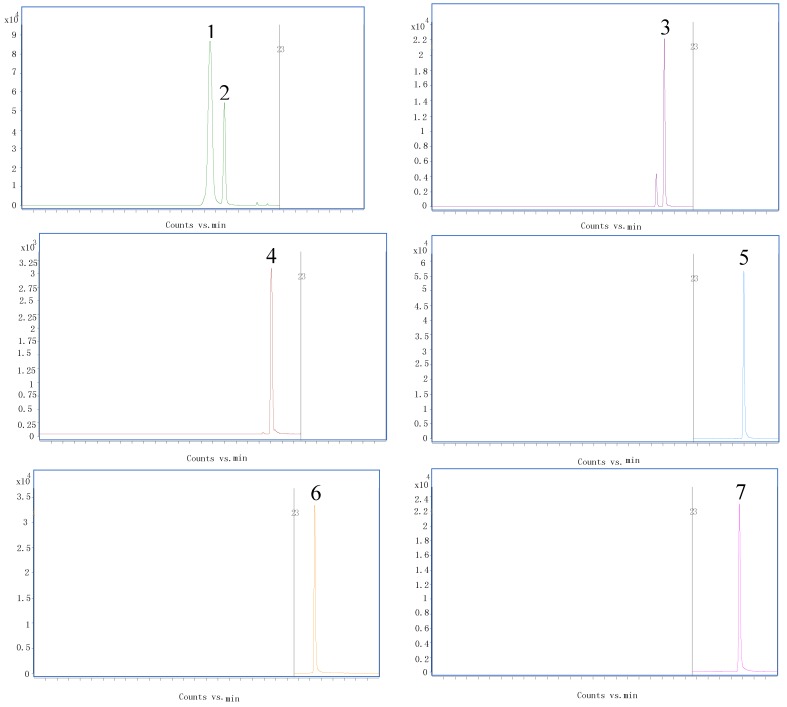
Chromatographic of flavonoid standards. Note: 1. Hyperoside 2. Isoquercitrin 3. Astragalin 4. Luteolin-7-*O*-glucoside 5. Isorhamnetin 6. Quercetin 7. Kaempferol.

**Figure 3:**
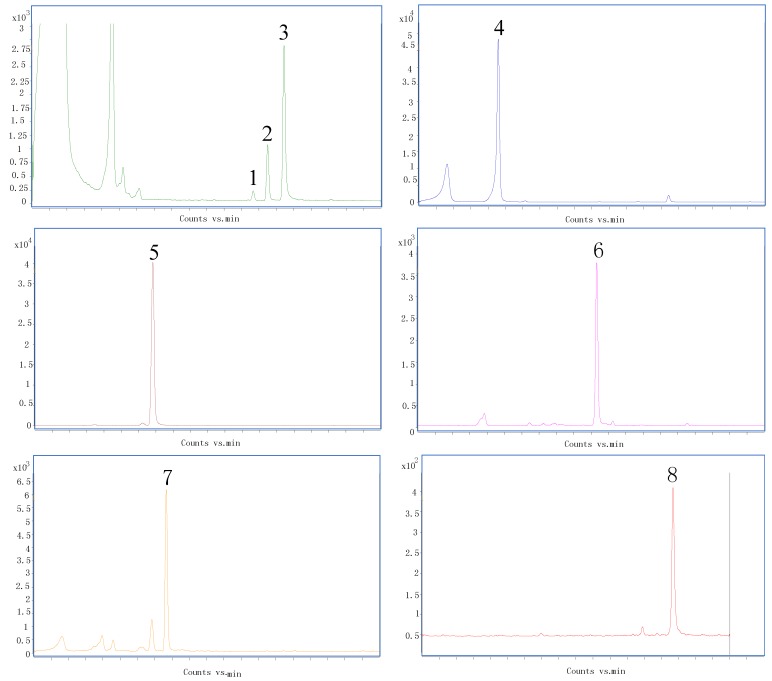
Chromatographic of chlorogenic acid standards. Note: 1. 3,4-dicaffeoylquinic acid 2. 3,5-dicaffeoylquinic acid 3. 4,5-dicaffeoylquinic acid 4. 3-caffeoylquinic acid 5. 4-caffeoylquinic acid 6. *p*-hydroxycinnamic acid 7. caffeic acid 8. 5-FQA.

Due to the lack of standards for some of the compounds, their negative identification was based on the correspondence of the ion from the deprotonated molecule with literature data, fragmentation patterns of other similar compounds and database. The chromatographic of standards were shown in Figure 2 and Figure 3. For the LC-MS measurements, negative ion mode was used to obtain the better tandem mass spectra and high-resolution mass spectra. In total of 45 compounds were identified, including 23 flavonoids, 2 lignans and 20 chlorogenic acids (Table 1). For all the compounds the high-resolution mass data was in good agreement with the theoretical molecular formulas, all displaying a mass error of below 5 ppm (Table 2) thus confirming their elemental composition.

### 2.4. Chlorogenic Acids

#### 2.4.1. Characterization of *p*-Coumaroylquinic Acids (*p*CoQA, Mr = 338.1002)

Chlorogenic acids (CGAs) are a family of esters of trans-cinnamic acids (most commonly p-coumaroyl, caffeoyl, feruloyl and dimethoxycinnamoyl acids) with quinic acid [9,10]. The trans-cinnamic acids can be esterified at one or more of the hydroxyls at positions 1, 3, 4, and 5 of quinic acid, originating series of positional isomers. More importantly, it is easy to distinguish a 4-acyl chlorogenic acids by its “dehydrated” MS^2^ base peak at *m*/*z* 173 ([quinic acid-H-H_2_O]^−^), supported by strong MS^3^ ions at *m*/*z* 93 [11,12]. *P*-Coumaroylquinic acid (*p*CoQA) has a molecular weight (Mr) of 338.1002 and three peaks (peak 34, 41, 42) at *m*/*z* 337 were detected. The three peaks are all *p*CoQA isomers. In addition, according to previous reported literature, the retention time of a 4-position substituted cis-isomer on a reverse phase chromatographic column is obviously longer than that of a trans-isomer [13]. Based on the above analysis, compounds 34 and 41 were identified as cis-4-*p*-CoQC and trans-4-*p*-CoQA, respectively. While compound 42 is *p*CoQA isomer which is uncertain.

#### 2.4.2. Characterization of Caffeoylquinic Acids (CQA, Mr = 354.0951)

**Figure 4:**
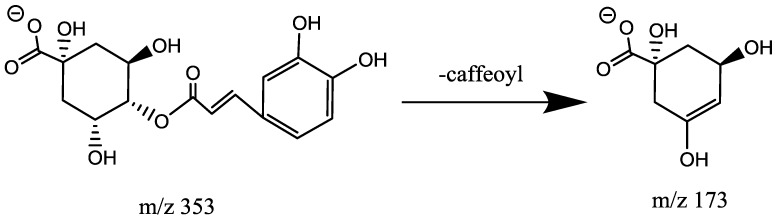
Fragmentation pathways of 4-*O*-caffeoylqunic acid.

**Figure 5:**
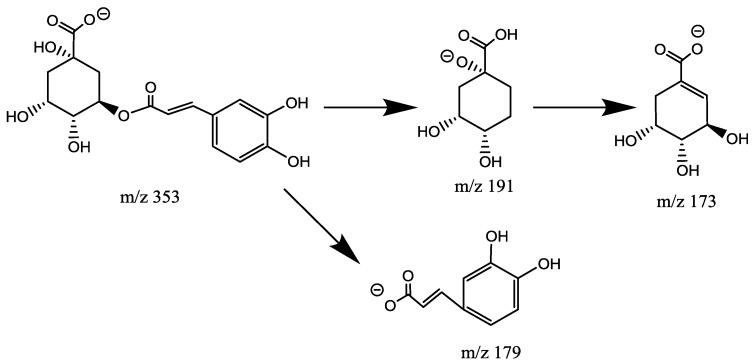
Fragmentation pathways of 3-*O*-caffeoylqunic acid.

Chromatographic peaks 13 and 32 presented *m*/*z* 353 as base peaks in negative ionization mode mass spectra, which suggested positional isomers of a quinic acid (QA) esterified with a single caffeoyl (CAF) unit. The product ion spectra obtained by negative ion MS/MS for precursor ions *m*/*z* 353 were different from each other. The product ion spectrum for peak 32 showed *m*/*z* 173 (dehydrated quinic moiety) as the base peak, *m*/*z* 191 [loss of caffeic moiety], and *m*/*z* 179 (loss of quinic moiety). As *m*/*z* 173 is a diagnostic ion that acylated at position 4, peak 32 was attributed to 4-CQA (Figure 4). As reported before, the retention times of acylated CQAs repeat the elution pattern: 3-acylquinic acid elutes first, followed by 4-acylquinic acids. So peak 13 is assumed as 3-CQA (Figure 5) [9,14].

#### 2.4.3. Characterization of Feruloylquinic Acids (FQA, Mr = 368.1107)

**Figure 6:**
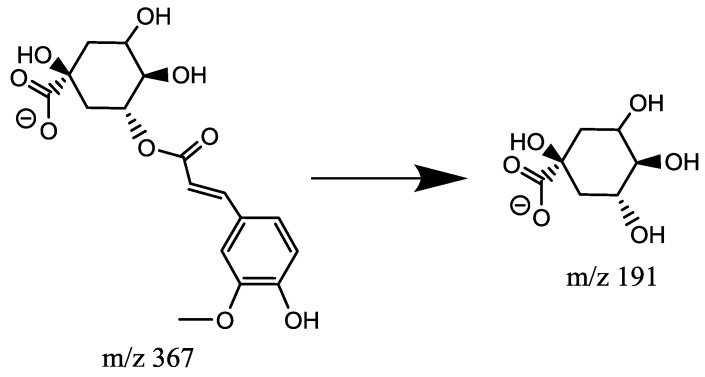
Fragmentation pathways of 5-FQA

Feruloylquinic acids has a Mr = 368.1107. Similar to *p*CoQA and CQA, molecules harboring ferulic acid moieties were also identified. The negative ionization mode fragmentation of the precursor ion *m*/*z* 367 of peak 19 produced *m*/*z* 191 as base peak. This is a diagnostic ion of acylation in position 5 of quinic acid (19) and allows the identification of the compound as 5-FQA (Figure 6), based on the chlorogenic acid identification by LEONARDO et al. [9]. 

#### 2.4.4. Characterization of Di-Caffeoylquinic Acid (di-CQA, Mr = 516.1268)

**Figure 7:**
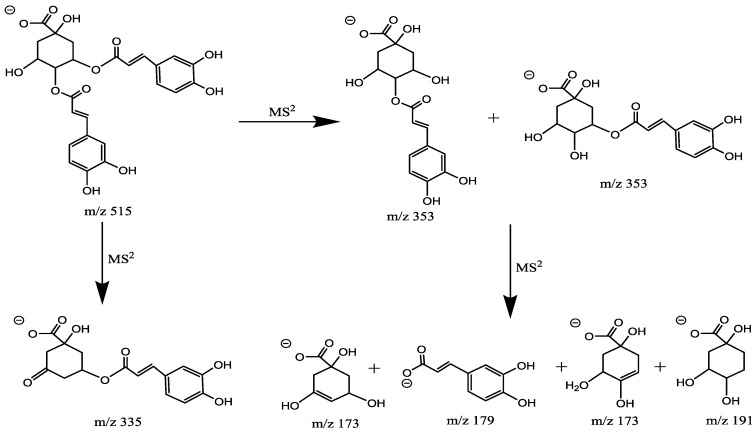
Fragmentation pathways of di-caffeoylquinic acid.

As previously reported, di-CQA produce an isobaric pseudomolecular ion at *m*/*z* 515. The diCQA are isomers as their chemical structures possess the same skeleton of quinic acid, and they can be differentiated by different substitution positions (Figure 7). Peaks 4 and 7 generated [M − H]^−^ ion at *m*/*z* 515 and [M − H-caffeoyl]^−^ ion at *m*/*z* 353 (the deprotonated molecular ions yielded via the neutral loss of 162 (C_9_H_6_O_3_)). Base peak in peak 6 at *m*/*z* 179, indicating it was 3-substituted quinic acid. This ion in 3, 4-diCQA (peaks 4) was at *m*/*z* 178 and in 4, 5-diCQA (peaks 7) was absent. 3, 5-DiCQA (peaks 6) was relatively easy to distinguish, owing to its MS^3^ base peak *m*/*z* 191 and similar intensities of ions at *m*/*z* 179 with data previously published [15]. Generally, it was observed that the order of elution for the diacyl CGAs in RP columns is 3, 4 > 3, 5 > 4, 5 [16]. By comparing with reference substances, peaks 4, 6 and 7 were assigned as 3, 4-diCQA, 3, 5-diCQA, 4, 5-diCQA which is consist with data previously published. 

#### 2.4.5. Characterization of Caffeoylglycoside (Mr = 342.0951)

Caffeoylglycoside have molecular weights of 342.0951 which indicates that the glucosyl group was linked to the caffeic acid, not quinic acid [13]. Two molecules (2 and 5) with pseudomolecular peaks at *m*/*z* 341 were assigned as isomers of caffeoylglycoside. They produced distinctive ions at *m*/*z* 179 ([caffeic acid-H]^−^) by the loss of a glucosyl residue (C_6_H_10_O_5_) and *m*/*z* 135 ([caffeic acid-H]^−^). As reported before, the retention time of a 6-position substituted *β*-glucose isomer on a reverse phase chromatographic column is obviously longer than that of *α*-glucose isomer [17]. On the basis of these arguments, the first eluting isomer was assigned as 6-*O*-caffeoyl-*β*-glucose (2) and the later eluting isomer as 6-*O*-caffeoyl-*α*-glucose (5).

#### 2.4.6. Characterization of Caffeoylquinic Acid Glucoside (CQA-Glycoside, Mr = 516.1434)

Caffeoylquinic acid glucoside has a Mr = 516.1434. Unlike the diCQA, the CQA glycosides showed a typical fragmentation pattern of chlorogenic acids. They produce distinctive ions which originated from the cinnamoyl glycoside part at *m*/*z* 341 (C_15_H_17_O_9_, [caffeoyl glucoside-H]^−^) or/and 323 (C_15_H_15_O_8_, [caffeoyl glucoside-H-H_2_O]^−^) which were not present in the diCQA MS spectra. Previous studies [14] led to the conclusion that CQA forms a glycoside through an ether bond at either C-3 or C-4 on the aromatic caffeoyl ring. However, a MS^2^ base peak at *m*/*z* 323 is a characteristic of glucosyl attachment at C-3 [18].

Peak 8 produced the MS^2^ base peak at *m*/*z* 341 ([caffeoyl glucoside-H]^−^) due to the loss of a quinic acid moiety (174 Da); the secondary peaks occurred as follows: the peak at *m*/*z* 353 ([caffeoyquinic acid-H]^−^) via the loss of a glucosyl residue (162 Da), the peak at *m*/*z* 179 ([caffeic acid-H]^−^) due to the loss of a glucosyl, the peak at *m*/*z* 191 ([quinic acid-H]^−^) due to the loss of a caffeoyl, a glucosyl residues and the peak at *m*/*z* 173 ([quinic acid-H_2_O-H]^−^) due to the loss of a caffeoyl and a glucosyl residues followed by H_2_O (18 Da) (Table 1).The MS^2^ spectrum was identical to 3-*O*-caffeoylquinic acid. The MS^2^ peaks at *m*/*z* 353 and 341 were absent which suggested that glucose was connected with the quinic acid moiety by an ether linkage and caffeic acid was connected with quinic acid at C-3 by an ester bond. This isomer is assigned as 3-*O*-(4′-*O*-caffeoyl glucosyl) quinic acid.

**Figure 8:**
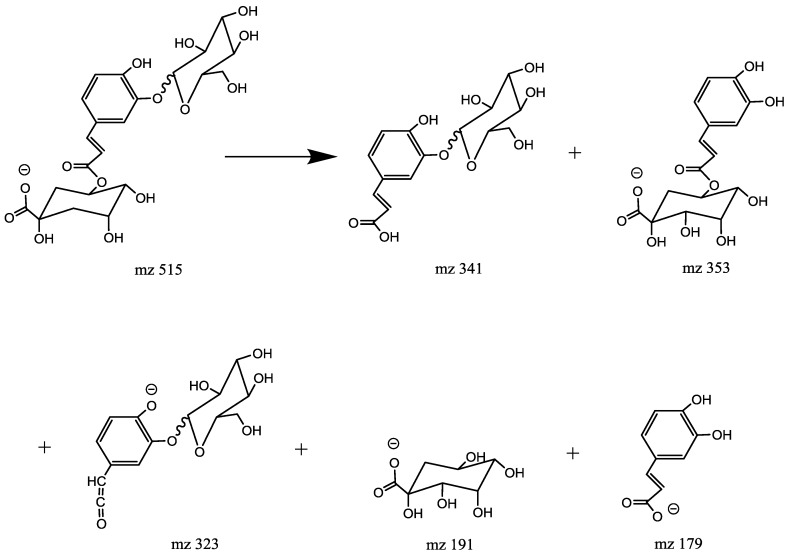
Fragmentation pathways of 5-*O*-(3′-*O*-Glucosylcaffeoyl) quinic acid.

Peak 10 produced the MS^2^ base peak at *m*/*z* 323 ([caffeoyl glucoside-H_2_O-H]^−^) due to the loss of a quinic acid moiety (174 Da) followed by H_2_O (18 Da) and secondary peaks at *m*/*z* 341 ([caffeoyl glucoside-H]^−^) due to the loss of a quinic acid moiety (174 Da), *m*/*z* 353 ([caffeoylquinic acid-H]^−^) via the loss of a glucosyl residue (162 Da), *m*/*z* 191 ([quinic acid-H]^−^) due to the loss of a caffeoyl. The MS^2^ spectra were identical to the MS^2^ spectra of 5-*O*-caffeoylquinic acid (Table 1). The MS^2^ peaks at *m*/*z* 353, 341 and 323 suggested that glucose was connected with the caffeic acid moiety by an ether linkage and caffeic acid was connected with quinic acid at C-5 by an ester bond [19]. From the above points it was clear that peak 10 can be identified as 5-*O*-(3′-*O*-Caffeoylglucosyl) quinic acid (Figure 8).

#### 2.4.7. Characterization of Coumaroyl-Tricaffeoylquinic Acid (Mr = 824)

Peak 18 exhibited [M − H]^−^ ion at *m*/*z* 823, which revealed *m*/*z* 661 by losing a caffeoyl moiety. Compounds 18 consequently lost a coumaroyl moiety and a caffeoyl moiety to produce [M − H-146-162]^−^ ion at *m*/*z* 353. It was tentatively identified as coumaroyl-tricaffeoylquinic acids.

### 2.5. Flavonoids Derivatives

An number of flavonoids have been reported from Cuscuta species previously. Most of them are flavonoids, flavonols and flavones glycoside [20]. 

#### 2.5.1. Characterization of Apigenin (Mr = 270.0528)

One peak was detected at *m*/*z* 269(peak 35) in the extracted ion chromatogram. It produced the MS^2^ base peak at *m*/*z* 225 corresponding to neutral loss of CO_2_ (44 Da). It was identified as apigenin, consistent with the literature data [20].

#### 2.5.2. Characterization of Isorhamnetin (Mr = 316.0583)

One peak was detected at *m*/*z* 315(peak 45) in the extracted ion chromatogram and was tentatively assigned as isorhamnetin. It produced the MS^2^ base peak at *m*/*z* 300 indicating the presence of a methoxyl group (loss of a methyl radical). These data matched those previously reported for isorhamnetin [20].

#### 2.5.3. Characterization of Kaempferol-Hexoside (Mr = 448.1006)

Four isomers were found at M/S 447(26, 27, 30 and 31). The four isomers yielded MS^2^ ion at *m*/*z* 285 as the base peak, indicating the existence of hexoside. Peak 26, 27 yielded fragment at *m*/*z* 285([A-H]^−^), *m*/*z* 255([A-H-HCHO]^−^) and *m*/*z* 227([A-H-CO]^−^) ion, consistent with the reported data for kaempferol [20]. As reported [20], peak 26 showed weaker retention on the RP-HPLC column than peak 27, therefore they were assigned as kaempferol-3-*O*-galactoside, astragalin respectively. For peak 31, there were no [A-H-30]^−^ ion, the base peak for MS^3^ was M/S 151 which is consist with luteolin, therefore peak 31 was identified as luteolin-7-*O*-glucoside and was confirmed by comparison with a reference standard. Peak 30 had similar fragments with peak 31, thus it was assigned as luteolin-hexoside.

#### 2.5.4. Characterization of Kaempferol-*O*-Dihexoside, Isorhamnetin-3-Apiosyl-(1→2)-Hexoside and Quercetin-3-*O*-Coumaroylgalactoside (Mr = 610.1323)

Peak 17, 24 and 37 all produced [M − H]^−^ ion at *m*/*z* 609. However, they produced obviously different MS^2^ ions. Peak 17 MS^2^ spectrum gave ions at *m*/*z* 447 and 285, originating from successive losses of 162 Da, suggesting the presence of two hexosyl residues. The [A-H]^−^ ion at *m*/*z* 285 yielded a [A-H-30]^−^ fragment at *m*/*z* 255, consistent with kaempferol. Thus, compound 17 was identified as kaempferol-*O*-dihexoside. Peak 24 exhibited a [M − H]^−^ ion at *m*/*z* 609. Its MS^2^ spectrum gave ions at *m*/*z* 315 and 300, originating from successive losses of 162 Da and 132 Da, suggesting the presence of hexosyl and apiosyl residues. The [A-H]^−^ ion at *m*/*z* 315 yielded a [A-H-15]^−^ fragment at *m*/*z* 300, consistent with isorhamnetin. Thus, peak 24 was assigned as isorhamnetin-3-apiosyl-(1**→**2)-hexoside. Peak 37 MS^2^ spectrum gave ions at *m*/*z* 463 and 301, originating from successive losses of 146 Da and 162 Da, suggesting the presence of hexosyl residue and coumaroyl residue. The product spectrum of the *m*/*z* 301 ion was very similar to that of quercetin, though no [A-H-30]^−^ ion was observed. Based on the fragmentation pattern, this compound was assigned as quercetin-3-*O*-coumaroylgalactoside. 

#### 2.5.5. Characterization of Isorhamnetin-Hexoside (Mr = 478.1111)

Two peaks were detected at *m*/*z* 477 in the extracted ion chromatogram and were tentatively assigned as isorhamnetin-hexoside (28 and 29). These two compounds produced base peak at *m*/*z* 314, originating from the loss of a hexose (162 Da), and MS^3^ spectrum was very similar to that of isorhamnetin. These compounds were thus tentatively identified as isorhamnetin-7-glucoside and isorhamnetin-3-*O*-glucoside, respectively.

#### 2.5.6. Characterization of Kaempferol-Glucoside (Mr = 568.1217)

For peak 33, a significant loss of 120 Da was also observed, but no direct loss of 162 Da from the [M − H]^−^ ion was observed. Therefore, it is rational to assign a *p*-hydroxybenzoyl group linked to the hexose moiety rather than the aglycone in this structure. Interestingly, a second loss of 120 Da (*m*/*z* 447→327) was also observed, which presumably results from ^1,2^X fragmentation of the hexose. Peak 33 produced MS^2^ base peak at *m*/*z* 285 whose fragmentation was consistent with kaempferol, and therefore peak 33 was finally confirmed as kaempferol-3-*O*-*p*-hydroxybenzoylglucoside, which is consistent with the previous report [20].

#### 2.5.7. Characterization of Kaempferol-*O*-Glucoside-7-Rhamnoside and Kaempferol-3-*O*-Coumaroylglucoside (Mr = 594.1373)

Peak 39, 40 displayed a [M − H]^−^ ion at *m*/*z* 593.The MS^2^ spectra of the reference substances kaempferol-3-*O*-glucoside-7-rhamnoside showed ion [M − H-146]^−^ that clearly indicates the removal of rhamnosyl moiety from the hydroxyl group of C7 and showed almost the same intensity as the aglycone. Previous studies [10] described the removal of the sugar residues from the hydroxyl in position 7 as being much more favored in ESI-MS than from position 3. Due to these findings compound 39 was identified as kaempferol-3-*O*-glucoside-7-rhamnoside.

Peak 40 displayed a [M − H]^−^ ion at *m*/*z* 593. The MS^2^ spectrum gave a base peak at *m*/*z* 285, originating from the concurrent losses of coumaroyl (146 Da) and a hexose (162 Da), which made of a disaccharide moiety. The *m*/*z* 447 ion resulted from the cleavage of the coumaroyl, which should thus be connected directly with the hexose moiety. The product spectrum of the *m*/*z* 285 ion was very similar to that of kaempferol, though no [A-H-30]^−^ ion was observed. Previous study [20] reported that the retention times of flavonoid diglycosides on RP-HPLC columns generally is longer than monoglycosides. Based on the above points, compound 40 was assigned as kaempferol-3-*O*-coumaroylglucoside.

#### 2.5.8. Characterization of Quercetin-3-*O*-Apiosyl-(1→2)-Galactoside (Mr = 596.1377)

One peak 20 was detected at *m*/*z* 595 in the extracted ion chromatogram and was tentatively assigned as quercetin-3-*O*-apiosyl-(1**→**2)-galactoside. CID of the [M − H]^−^ ion gave two major ions at *m*/*z* 463 and 301, consistent with successive losses of apiose (132 Da) and galactose (162 Da). Similar to astragalin, daughter ion at *m*/*z* 301 produced a [A-H-30]^−^ ion at *m*/*z* 271, thus conforming it as a quercetin glycoside. The ^1,2^A^−^ ion at *m*/*z* 179 was also observed, in agreement with the literature data [20].

#### 2.5.9. Characterization of Kaempferol-3-*O*-Galactoside and Quercetin-Hexoside (Mr = 464.0955)

Peak 21 gave MS^2^ and MS^3^ spectra very similar to those of astragalin, and was plausibly identified as hyperoside, which has been previously reported [20]. However peak 22 had similar fraction information with 21, so it was tentatively identified as isoquercitrin.

#### 2.5.10. Characterization of Quercetin-3-(2′′-Acetylgalactoside) (Mr = 506.1060)

Peak 25 exhibited [M − H]^−^ ion at *m*/*z* 505, which yielded an MS^2^ base peak at *m*/*z* 301 by losing a *m*/*z* 204 moiety which is identified as acetylhexose [21]. The base peak at *m*/*z* 301 indicates that hexose group is connected with flavonoid. *M*/*z* 301 yielded fragment at *m*/*z* 179 and 151, consist with quercetin. Peak 25 was identified as quercetin 3-(2′′-acetylgalactoside), which is consistent with the previous report [20].

#### 2.5.11. Characterization of Quercetin-Dihexoside (Mr = 626.1483)

Quercetin-3-*O*-galactoside-7-*O*-glucoside (peak 11) exhibited base peak at *m*/*z* 463, originating from the loss of hexose (162 Da). Fragment at *m*/*z* 301 was consequently the successive loss of hexose (162 Da). The fragmentation pattern was identical to previously studied data [20]. Similarly, peak 11 was assigned as quercetin-dihexoside.

#### 2.5.12. Characterization of Kaempferol 3-Apiosyl-(1→2)-Glucoside (Mr = 580.1428)

One peak was detected at *m*/*z* 579 in the extracted ion chromatogram and was tentatively assigned as kaempferol-3-apiosyl-(1**→**2)-glucoside (23). Apiose was the only pentose hitherto reported in flavonoid glycosides of Cuscuta species, and could be characterized by a 132 Da loss [20]. Compound 23 produced base peak at *m*/*z* 285 via the loss of pentose and hexose residues which indicated that hexose was connected with pentose residue. Fragment ion at *m*/*z* 285 yielded a [A-H-30]^−^ fragment at *m*/*z* 255, consistent with kaempferol. Thus, compound 23 was identified as kaempferol-3-apiosyl-(1**→**2)-glucoside. Furthermore, peak 23 is its geometric isomer.

Chemical constituents in Semen Cuscutae were analyzed by LC/MS. From the structural characterization by HPLC-MS, 45 compounds were identified based on their retention behavior. As a result, 45 compounds including 20 chlorogenic acids, 23 flavonoids and 2 lignans were identified based on their retention behavior. 23 of them are reported for the first time including 6-*O*-caffeoyl-*β*-glucose and 3-*O*-(4′-*O*-Caffeoylglucosyl) quinic acid and so on (Table 2).

## 3. Materials and Methods 

### 3.1. Materials and Chemicals

**Figure 9:**
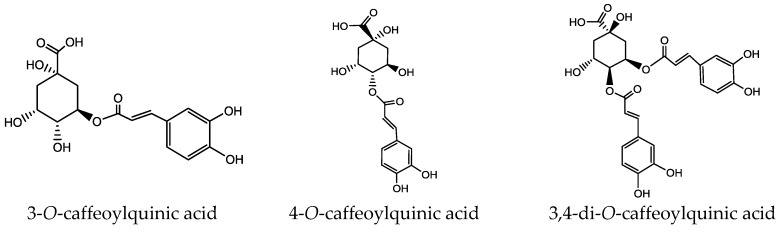

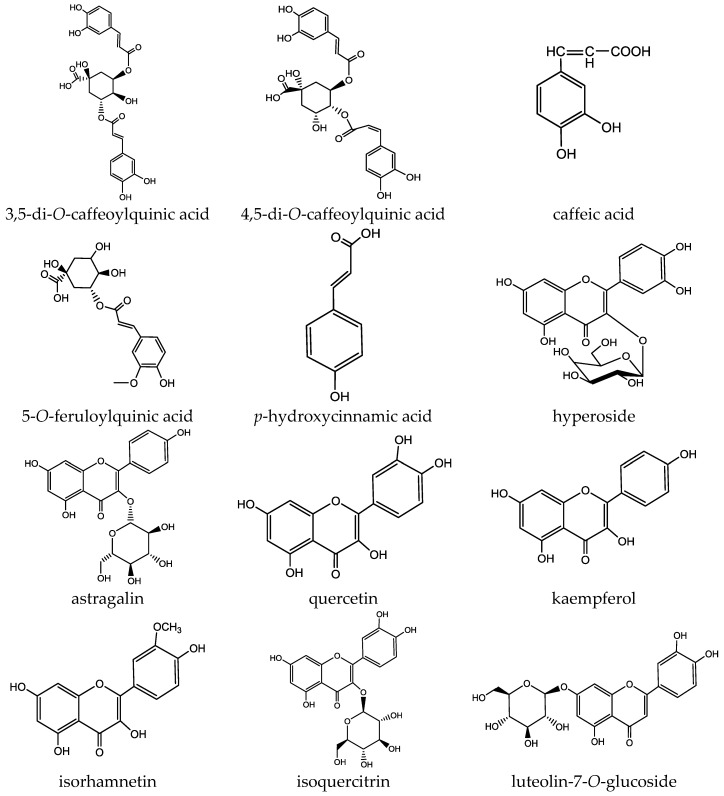
Chemical formula of flavonoids of Semen Cuscutae.

Five flavonoid reference standards including hyperoside, quercetin, astragalin, kaempferol, and isorhamnetin were purchased from National Institutes for Food and Drug Control (Beijing, China). Two flavonoid reference standards including isoquercitrin and luteolin-7-*O*-glucoside were purchased from Shanghai Yuanye Bio-Technology Co., Ltd. (Shanghai, China). Eight chlorogenic acid reference standards including 3-caffeoylquinic acid(3-CQA), 4-caffeoylquinic acid(4-CQA), 3,4-dicaffeoylquinic acid (3,4-DiCQA), 3,5-dicaffeoylquinic acid(3,5-DiCQA), 4,5-dicaffeoylquinic acid(4,5-DiCQA), *p*-hydroxycinnamic acid, caffeic acid(CA) and 5-*O*-feruloylquinic acid (5-FQA) were purchased from Shanghai Yuanye Bio-Technology Co., Ltd. (Shanghai, China). Their structures (shown in Figure 9) were fully elucidated by spectra data (ESI-MS). The purities of all the standards were no less than 98%. All the standard were resolved in 80% methanol.

Acetonitrile (MS grade) and formic acid (MS grade) were purchased from Thermo Fisher Scientific Inc. Deionized water used throughout the experiment was purified by MilliQ50 SP Reagent Water System (Bedford, MA, USA) for preparing samples.

### 3.2. Sample Collection

The crude products of Semen Cuscutae (Lot number: 160161211) were purchased from Beijing Kangmei Pharmaceutical Co., Ltd. which were identified and authenticated as Semen Cuscutae by Yang Yaojun, the professor of Pharmacognosy Department in Beijing University of Chinese Medicine. Voucher specimens were retained in the School of Chinese Materia, Beijing University of Chinese Medicine.

### 3.3. Extraction Method

The extraction method referenced to our previous study and was set as follow [5]: powdered samples (60 mesh, 1 g) were suspended in 80% methanol (50 mL) and extracted under reflux for 2 h. After cooling, the loss of weight was replenished with 80% methanol. All solvents and samples were filtered through 0.22-μm organic-membranes prior to injection.

### 3.4. UPLC−MS Analysis

The extracts were chromatographically separated on an ACQUITY UPLC BEH C_18_ column (2.1 × 100 mm i.d., 1.7 μm). The mobile phase consisted of A (acetonitrile) and B (water containing 0.05% formic acid, *v*/*v*). The flow rate was 0.20 mL/min. The elution conditions applied with a linear gradient as follows: 0–4 min, 7–16% A; 4–8 min, 16–17% A; 8–15 min, 17–17% A; 15–20 min, 17–24% A; 20–27 min, 35–52% A; 27–33 min, 52–97% A. Column temperature was 35 °C.

For LC/MS analysis, an LTQ-Orbitrap mass spectrometer (Thermo Scientific, Bremen, Germany) was connected to the Ultra-High-Performance Liquid Chromatography instrument via an electrospray ionization (ESI) interface. Samples were analyzed in negative ion mode with a tune method set as follows: sheath gas (nitrogen) flow rate of 40 arb, aux gas (nitrogen) flow rate of 20 arb, source voltage, 4 kV, capillary temperature of 350 °C, capillary voltage of 25 V, and tube lens voltage of −110 V. Accurate mass analysis was calibrated according to the manufacturer’s guidelines. Centroided mass spectra were acquired in mass range of *m*/*z* 50–1000 and resolution set at 30,000 using a normal scan rate detected by Orbitrap analyzer.

### 3.5. Data Processing

Thermo Xcaliber 2.1 (Thermo Fisher Scientific, San Jose, CA, USA) was used for qualitative data acquiring and processing. All the relevant data including peak number, retention time, accurate mass and predicted chemical formula were recorded into an Excel file.

## 4. Conclusions

In this study, we identified 45 compositions in Semen Cuscutae using UPLC coupled with electrospray ionization tandem mass spectrometry system. 23 of them are reported for the first time including 6-*O*-caffeoyl-*β*-glucose, 3-*O*-(4′-*O*-Caffeoylglucosyl) quinic acid, etc. As chlorogenic acids and flavonoids are phenolic compounds which are predominant compounds in Semen Cuscutae, we can conclude that phenolic compounds are the major constituents of Semen Cuscutae.

## Figures and Tables

**Table 1:** Compounds identified in Semen Cuscutae.

No.	t_R_(min)	Identification	[M − H] or [M+FA-H]	Molecular Formula	Exact Mass	[M − H]-*m*/*z*	Characteristic *m*/*z* of Ions in Negative Ion Mode
1	1.18	Caffeoyl glucoside	M+FA-H			539.1653	MS^2^:503(100), 341(73) MS^3^:179(100), 323(25), 341(10)
2	1.31	6-*O*-caffeoyl-*β*-glucose	M − H	C_15_H_18_O_9_	342.0951	341.1068	MS^2^:179(100), 281(2), 221(1) MS^3^:135(100)
3	1.32	Caffeoyl diglucoside	M − H			503.1590	MS^2^:179(100), 221(51), 323(22), 161(15), 341(15) MS^3^:161(100),143(63), 131(14)
4	1.46	3,4-diCQA	M − H	C_25_H_24_O_12_	516.1268	515.1378	MS^2^:353(100), 191(62), 178(9), 173(2)
5	1.58	6-*O*-Caffeoyl-*α*-glucose	M − H	C_15_H_18_O_9_	342.0951	341.1075	MS^2^:179(100), 281(2) MS^3^:135(100)
6	2.34	3,5-diCQA	M − H	C_25_H_24_O_12_	516.1268	515.1400	MS^2^:179(100), 353(69), 191(22), 335(3), 173(3), MS^3^:191(100), 179(10), 135(4)
7	2.93	4,5-diCQA	M − H	C_25_H_24_O_12_	516.1479	515.1403	MS^2^:353(100), 191(63), 179(2) MS^3^:191(100), 179(5)
8	4.01	3-*O*-(4′-*O*-Caffeoylglucosyl)quinic acid	M − H	C_22_H_28_O_14_	516.1434	515.1397	MS^2^:341(100), 353(87), 191(4), 173(41), 179(57)
9	4.04	*p*-Hydroxycinnamic acid	M − H	C_9_H_8_O_3_	164.0473	163.0398	MS^2^:119(100) MS^3^:119(100), 75(46)
10	4.73	5-*O*-(3′-*O*-Caffeoylglucosyl)quinic acid	M − H	C_22_H_28_O_14_	516.1479	515.1396	MS^2^:323(100), 191(26), 353(24), 341(16), 179(3)
11	5.35	Quercetin-3-*O*-galactoside-7-*O*-glucoside	M − H	C_27_H_30_O_17_	626.1483	625.1390	MS^2^:463(100),505(3), 301(2) MS^3^:301(100), 179(1)
12	5.47	Quinic acid	M − H	C_7_H_12_O_6_	192.0634	191.0553	MS^2^:127(100), 85(86), 173(63), 93(58) MS^3^:85(100), 99(40), 109(27)
13	5.51	3-*O*-Caffeoylqunic acid	M − H	C_16_H_18_O_9_	354.0951	353.0856	MS^2^:191(100), 179(3) MS^3^:127(100), 173(76)
14	5.85	Caffeic acid	M − H	C_9_H_8_O_4_	180.0423	179.0347	MS^2^:135(100), 107(27) MS^3^:107(100), 78(68)
15	6.01	Quercetin-3-*O*-caffeoylgalactoside	M − H	C_27_H_30_O_17_	626.1483	625.1396	MS^2^:463(100), 301(33), 505(5) MS^3^:301(100), 343(10)
16	6.48	Coumaroyl caffeoylglycoside	M − H	C_24_H_24_O_11_	488.1319	487.1449	MS^2^:265(100), 163(85), 307(71), 235(60), 145(53), 325(30), 341(15), 323(6)
17	6.58	Kaempferol-dihexoside	M − H	C_27_H_30_O_16_	610.1534	609.1452	MS^2^:447(100), 285(16) MS^3^:284(100), 285(52), 327(22), 255(14)
18	7.56	Coumaroyl-tricaffeoylquinic acid	M − H			823.2269	MS^2^:661(100) MS^3^:487(100), 353(94), 515(34),
19	7.73	5-FQA	M − H	C_17_H_20_O_9_	368.1107	367.1025	MS^2^:191(100), 173(100) MS^3^:127(100), 85(83), 173(81), 93(46), 111(36)
20	9.01	Quercetin-3-*O*-apiosyl-(1→2)-galactoside	M − H	C_26_H_28_O_16_	596.1377	595.1292	MS^2^:300(100), 301(48), 463(24) MS^3^:271(100), 255(58), 179(1)
21	10.03	Hyperoside	M − H	C_21_H_20_O_12_	464.0955	463.0860	MS^2^:301(100), 300(33), 343(4) MS^3^:179(100), 151(79), 273(18), 257(12)
22	10.40	Isoquercitrin	M − H	C_21_H_20_O_12_	464.0955	463.0866	MS^2^:301(100) MS^3^:179(100), 151(91), 273(16), 255(9)
23	10.65	Kaempferol-3-apiosyl-(1→2)-glucoside	M − H	C_26_H_28_O_15_	580.1428	579.1344	MS^2^:285(100), 284(60), 447(24), 255(15) MS^3^:257(100), 151(69), 241(46)
24	11.50	Isorhamnetin-3-apiosyl-(1→2)-hexoside	M − H	C_27_H_30_O_16_	610.1534	609.1448	MS^2^:315(100), 314(29), 300(22), 459(13) MS^3^:300(100), 287(5)
25	11.86	Quercetin 3-(2′′-acetylgalactoside)	M − H	C_23_H_22_O_13_	506.1060	505.0979	MS^2^:301(100), 300(53), 463(23) MS^3^:179(100), 151(89)
26	12.04	Kaempferol-3-*O*-galactoside	M − H	C_21_H_20_O_11_	448.1006	447.0926	MS^2^:284(100), 285(61), 327(14) MS^3^:255(100), 227(9)
27	13.29	Astragalin	M − H	C_21_H_20_O_11_	448.1006	447.0927	MS^2^:284(100), 285(58), 327(12) MS^3^:255(100), 256(17), 227(13)
28	13.42	Isorhamnetin-7-glucoside	M − H	C_22_H_22_O_12_	478.1111	477.1029	MS^2^:314(100), 315(71), 357(18), 449(8) MS^3^:285(100), 271(74), 300(34), 243(27)
29	14.31	Isorhamnetin-3-*O*-glucoside	M − H	C_22_H_22_O_12_	478.1111	477.1033	MS^2^:314(100), 315(36), 285(9), 300(3) MS^3^:285(100), 271(77), 300(35), 243(25),
30	14.4	Luteolin-hexoside	M − H	C_21_H_20_O_11_	448.1006	447.0927	MS^2^:285(100), 327(5) MS^3^:285(100), 151(32), 257(20), 229(10), 241(20)
31	16.12	Luteolin-7-*O*-glucoside	M − H	C_21_H_20_O_11_	448.1006	447.0926	MS^2^:285(100), 284(13), 327(5), 257(2) MS^3^:151(100), 257(32), 241(22)
32	16.73	4-*O*-Caffeoylqunic acid	M − H	C_16_H_18_O_9_	354.0951	353.0871	MS^2^:173(100), 179(47), 191(16), 135(7) MS^3^:93(100), 111(77)
33	21.23	Kaempferol-3-*O*-*p*-hydroxybenzoylglucoside	M − H	C_28_H_24_O_13_	568.1217	567.1135	MS^2^:284(100), 285(97), 447(37), 255(30), 429(11), 327(9) MS^3^:285(100), 151(6)
34	22.17	*cis*-4-*p*CoQA	M − H	C_16_H_18_O_8_	338.1002	337.0922	MS^2^:173(100), 163(9) MS^3^:93(100), 111(58)
35	22.76	Apigenin	M − H	C_15_H_10_O_5_	270.0528	269.0928	MS^2^:225(100)
36	23.23	Cuscutoside D	M − H	C_37_H_46_O_21_	826.2532	825.2439	MS^2^:369(100), 663(76), 323(43) MS^3^:219(100), 339(74), 311(54), 323(11)
37	23.32	Quercetin-3-*O*-coumaroylgalactoside	M − H	C_30_H_26_O_14_	610.1323	609.1242	MS^2^:463(100), 301(13) MS^3^:301(100), 343(3)
38	23.75	Quercetin	M − H	C_15_H_10_O_7_	302.0427	301.0349	MS^2^:151(100), 179(97)
39	24.69	Kaempferol-3-*O*-glucoside-7-rhamnoside	M − H	C_30_H_26_O_13_	594.1373	593.1289	MS^2^:447(13), 285(100), 327(1) MS^3^:257(81),285(100), 151(63), 241(45), 229(3)
40	25.17	Kaempferol-3-*O*-coumaroylglucoside	M − H	C_30_H_26_O_13_	594.1373	593.1290	MS^2^:285(100), 447(12), 307(6) MS^3^:285(100), 257(70), 151(59), 229(36), 241(35)
41	25.21	*trans*-4-*p*CoQA	M − H	C_16_H_18_O_8_	338.1002	337.0923	MS^2^:173(100), 163(8) MS^3^:93(100), 111(57)
42	25.74	*p*CoQA isomer	M − H	C_16_H_18_O_8_	338.1002	337.0923	MS^2^:173(100), 322(57), 306(14) MS^3^:93(100), 111(62), 155(36),
43	27.12	Cuscutoside A	M − H			663.1917	MS^2^:369(100) MS^3^:219(100), 339(77), 311(45)
44	27.77	Kaempferol	M − H	C_15_H_10_O_6_	286.0477	285.0392	MS^2^:285(100), 229(17)
45	28.54	Isorhamnetin	M − H	C_16_H_12_O_7_	316.0583	315.0502	MS^2^:300(100) MS^3^:271(100), 151(91), 272(62), 255(30)

**Table 2:** The existence of each component in Semen Cuscutae.

No.	t_R_(min)	Compound	Molecular Formula	New or Not	Mass Error (ppm)
**1**	1.18	Caffeoyl glucoside		+	4
**2**	1.31	6-*O*-caffeoyl-*β*-glucose	C_15_H_18_O_9_	+	2
**3**	1.32	Caffeoyl diglucoside		+	3
4	1.46	3,4-diCQA	C_25_H_24_O_12_	-	1
**5**	1.58	6-*O*-Caffeoyl-α-glucose	C_15_H_18_O_9_	+	4
6	2.34	3,5-diCQA	C_25_H_24_O_12_	-	1
7	2.93	4,5-diCQA	C_25_H_24_O_12_	-	1
**8**	4.01	3-*O*-(4′-*O*-Caffeoylglucosyl)quinic acid	C_22_H_28_O_14_	+	3
9	4.04	*p*-Hydroxycinnamic acid	C_9_H_8_O_3_	-	1
**10**	4.73	5-*O*-(3′-*O*-Caffeoylglucosyl)quinic acid	C_22_H_28_O_14_	+	4
11	5.35	Quercetin-3-*O*-galactoside-7-*O*-glucoside	C_27_H_30_O_17_	-	3
12	5.47	Quinic acid	C_7_H_12_O_6_	-	4
13	5.51	3-*O*-Caffeoylqunic acid	C_16_H_18_O_9_	-	2
**14**	5.85	Caffeic acid	C_9_H_8_O_4_	+	1
15	6.01	Quercetin-3-*O*-caffeoylgalactoside	C_27_H_30_O_17_	-	2
**16**	6.48	Coumaroyl caffeoylglycoside	C_24_H_24_O_11_	+	4
17	6.58	Kaempferol-dihexoside	C_27_H_30_O_16_	-	1
18	7.56	Coumaroyl-tricaffeoylquinic acid		-	3
**19**	7.73	5-FQA	C_17_H_20_O_9_	+	3
20	9.01	Quercetin-3-*O*-apiosyl-(1→2)-galactoside	C_26_H_28_O_16_	-	2
21	10.03	Hyperoside	C_21_H_20_O_12_	-	4
22	10.40	Isoquercitrin	C_21_H_20_O_12_	-	4
**23**	10.65	Kaempferol-3-apiosyl-(1→2)-glucoside	C_26_H_28_O_15_	+	1
**24**	11.50	Isorhamnetin-3-apiosyl-(1→2)-hexoside	C_27_H_30_O_16_	+	2
**25**	11.86	Quercetin 3-(2′′-acetylgalactoside)	C_23_H_22_O_13_	+	1
26	12.04	Kaempferol-3-*O*-galactoside	C_21_H_20_O_11_	-	1
27	13.29	Astragalin	C_21_H_20_O_11_	-	1
28	13.42	Isorhamnetin 7-glucoside	C_22_H_22_O_12_	-	1
**29**	14.31	Isorhamnetin-3-*O*-glucoside	C_22_H_22_O_12_	+	1
**30**	14.40	Luteolin-hexoside	C_21_H_20_O_11_	+	1
**31**	16.12	Luteolin-7-*O*-glucoside	C_21_H_20_O_11_	+	1
32	16.73	4-*O*-Caffeoylqunic acid	C_16_H_18_O_9_	-	1
33	21.23	Kaempferol-3-*O*-*p*-hydroxybenzoylglucoside		-	1
**34**	22.17	*cis*-4-*p*CoQA	C_16_H_18_O_8_	+	2
35	22.76	Apigenin	C_15_H_10_O_5_	-	1
36	23.23	Cuscutoside D	C_37_H_46_O_21_	-	1
37	23.32	Quercetin-3-*O*-coumaroylgalactoside	C_30_H_26_O_14_	-	1
38	23.75	Quercetin	C_15_H_10_O_7_	-	1
39	24.69	Kaempferol-3-*O*-glucoside-7-rhamnoside	C_30_H_26_O_13_	-	2
**40**	25.17	Kaempferol-3-*O*-coumaroylglucoside	C_30_H_26_O_13_	+	1
**41**	25.21	*trans*-4-*p*CoQA	C_16_H_18_O_8_	+	2
**42**	25.74	*p*CoQA isomer	C_16_H_18_O_8_	+	2
43	27.12	Cuscutoside A		-	1
44	27.77	Kaempferol	C_15_H_10_O_6_	-	4
45	28.54	Isorhamnetin	C_16_H_12_O_7_	-	2

Note: + represents a newly discovered compound in Semen Cuscutae, - represents the existing compound.
